# Tracking stolen bikes in Amsterdam

**DOI:** 10.1371/journal.pone.0279906

**Published:** 2023-02-15

**Authors:** Titus Venverloo, Fábio Duarte, Tom Benson, Pietro Leoni, Serge Hoogendoorn, Carlo Ratti

**Affiliations:** 1 Senseable City Lab, Massachusetts Institute of Technology, Cambridge, MA, United States of America; 2 Faculty of Civil Engineering and Geosciences (CiTG), Transport & Planning Department, Delft University of Technology, Delft, The Netherlands; The University of Tokyo, JAPAN

## Abstract

Crime has major influences in urban life, from migration and mobility patterns, to housing prices and neighborhood liveability. However, urban crime studies still largely rely on static data reported by the various institutions and organizations dedicated to urban safety. In this paper, we demonstrate how the use of digital technologies enables the fine-grained analysis of specific crimes over time and space. This paper leverages the rise of ubiquitous sensing to investigate the issue of bike theft in Amsterdam—a city with a dominant cycling culture, where reportedly more than 80,000 bikes are stolen every year. We use active location tracking to unveil where stolen bikes travel to and what their temporal patterns are. This is the first study using tracking technologies to focus on two critical aspects of contemporary cities: active mobility and urban crime.

## Introduction

Cities have been turning towards active modes of mobility to deal with increasing congestion, worsening air quality, and decreasing quality of life [1]. One example is the worldwide implementation of bike-sharing systems, which have shown to bring environmental and health benefits [2,3]. Similar benefits stem from the increasing use of bikes, with cities expanding and improving cycling infrastructure [4]—which for instance led to a 3.5-fold increase of cyclists in Lisbon [5]. Yet with the increased use of bikes, there is also an increase in bicycle thefts—which in turn discourages bicycle use [6].

One of the major hurdles to tackling bike theft is that it is typically seen as a low police priority, and that it is not addressed systematically [7]. Citizens, police and other governmental institutions see bike theft as incidental, making it difficult to see the aggregate picture. Yet the issue is relevant for society: for example, in the Netherlands alone the value of the stolen bike market is estimated at around 600 million euros annually [8].

In contrast to previous studies utilizing static crime data or prevention measures against bike theft [9–11], in this paper we developed a novel methodology to investigate bike theft using low-cost GPS-trackers to trace what happens with stolen bikes in Amsterdam. We do this by 1. determining the factors that determine likelihood of a bike being stolen; and 2. developing a technique to investigate stolen bike routes. By finding patterns of when and where bikes are stolen, and what happens with them, this research might aid in reducing bike theft, and also demonstrate the applicability of emerging sensing capabilities to the field of urban criminology.

The paper first presents a short literature review about bike theft, in which knowledge gaps are identified. Based on the identified gaps, the developed methodology is introduced, after which the results, discussion, and conclusions are presented.

### Literature review: Bike theft

Investigating bike theft in Washington, DC, Levy, Irvin-Erickson and La Vigne [11] find that thefts occur more frequently around metro stations. This is because stations have a high number of targets (as many bikes are parked there), there are many possible offenders passing through and mingling with the crowd, and there are fewer visitors during the night, resulting in less guardianship—a combination of factors that increases the likelihood of high rates of bike theft.

Zhang, Messner and Liu [9] observe that due to increased bicycle ownership, poor registration systems and a lack of legal protection in China, bike theft has increased significantly. As this is also a conclusion from Van Dijk et al [12] and Kuppens et al [8], the similarities on this issue between vastly different countries are noteworthy. Chen, Liu and Sun [13(p6)] summarize this quite well: “From the perspective of the offenders, bicycles are attractive objects. They are widely available, easy to steal, use, and resell, and difficult to track”.

As biking is becoming a more attractive mode of transport in cities, with more biking infrastructure being implemented every year, bike theft could hinder bike adoption—a central measure in cities fostering healthier and more sustainable modes of transportation. The current lack of systematic reporting, monitoring and mapping of when and where bikes are stolen, where bikes are taken to, whether it is used by the thief or is sold in an informal market, is a black box—and it might not only hinder bike adoption but also indicate a broader urban crime network.

In the Netherlands, a country well known for its high bicycle ridership, 466,000 bikes were estimated to be stolen in 2019—down from 560,000 in 2017 [8]. However, the average price of a stolen bike has increased. As such the stolen bike market of an estimated 600 million euros in the Netherlands alone remains a very large, somewhat neglected problem [8]. Furthermore, as one of the most frequently committed crimes in the Netherlands, bike theft has relatively low legal repercussions and the chances of arrest are very slim with scarce police resources for this issue [8].

Similar to what happens in other countries, train stations, shopping areas, and city squares are identified as hotspots of bike theft, with an additional upward trend in the number of bikes stolen from garages and gardens [14]. Secondary hotspots can be identified along the borders of the Netherlands; and experts interviewed by Kuppens et al [8] indicate that these bikes are often transported to eastern Europe.

**Fig 1:**
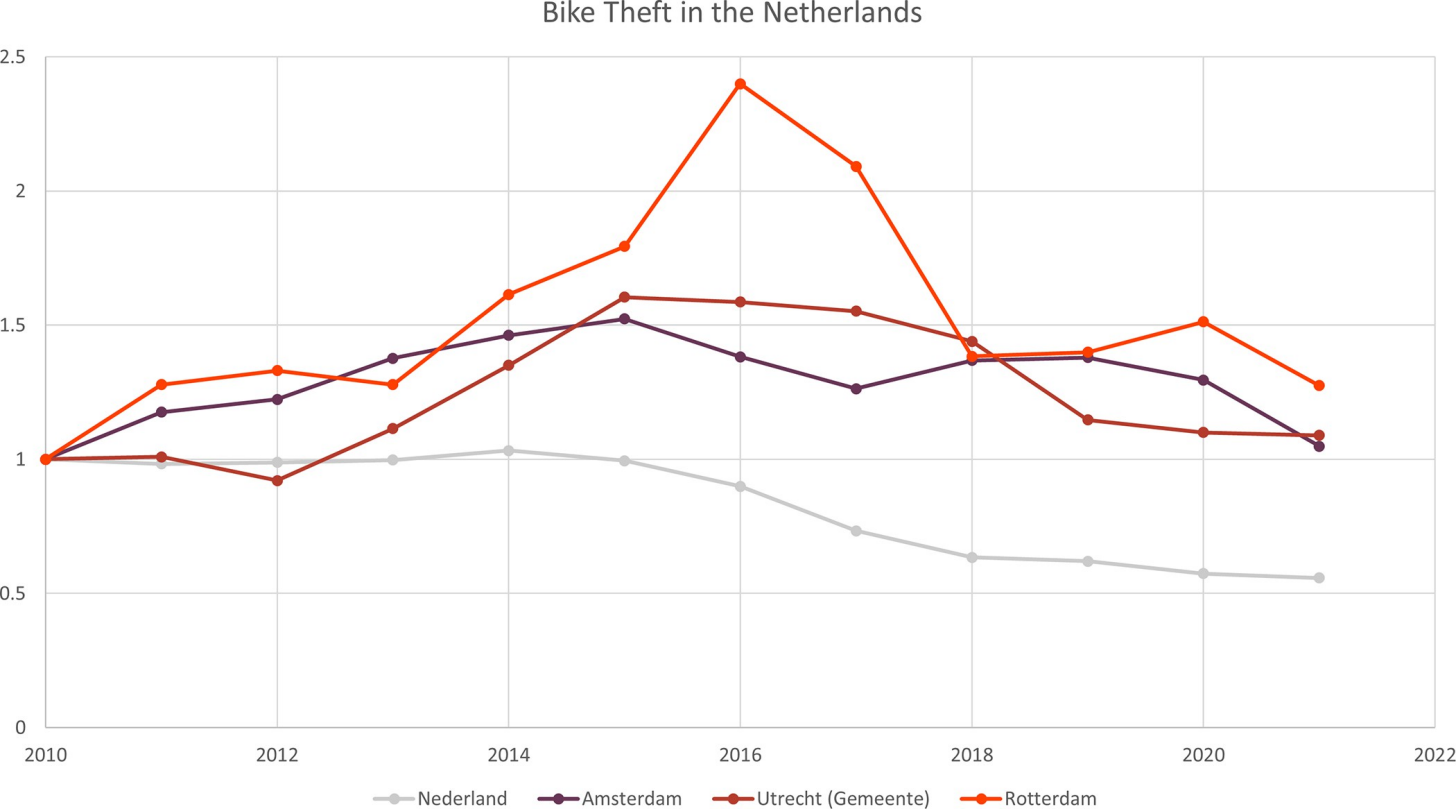
Reported bike thefts in the Netherlands, Amsterdam, Utrecht (municipality), and Rotterdam (2010 = 1). Data from 2020 and 2021 are preliminary. Data by [15].

The capital of the Netherlands, Amsterdam, has the most bikes stolen out of all Dutch cities; and while nationally the number of stolen bikes is decreasing, in Amsterdam and other larger Dutch cities it has increased or remained at a steady level of reported thefts (Fig 1).

In Amsterdam, approximately 11,000 bikes are reported stolen per year, although the municipality estimates the actual number around 28,500 bikes, and bike advocacy groups state the number is closer to 80,000. The difference can be explained by various measurements of the willingness to report bike theft to the local authorities. The municipality considers that 40% of the victims of bike theft report it [16], while Kuppens et al [8] found that in 2012, only 17.1% of the people in the Netherlands reported bike theft, decreasing to 14.2% in 2019.

The regional safety monitor of Amsterdam even indicates that in 2019 the number of residents who experienced bike theft was 18%, which would imply that the total number of bike thefts per year in Amsterdam may far exceed 100,000 [17].

**Fig 2:**
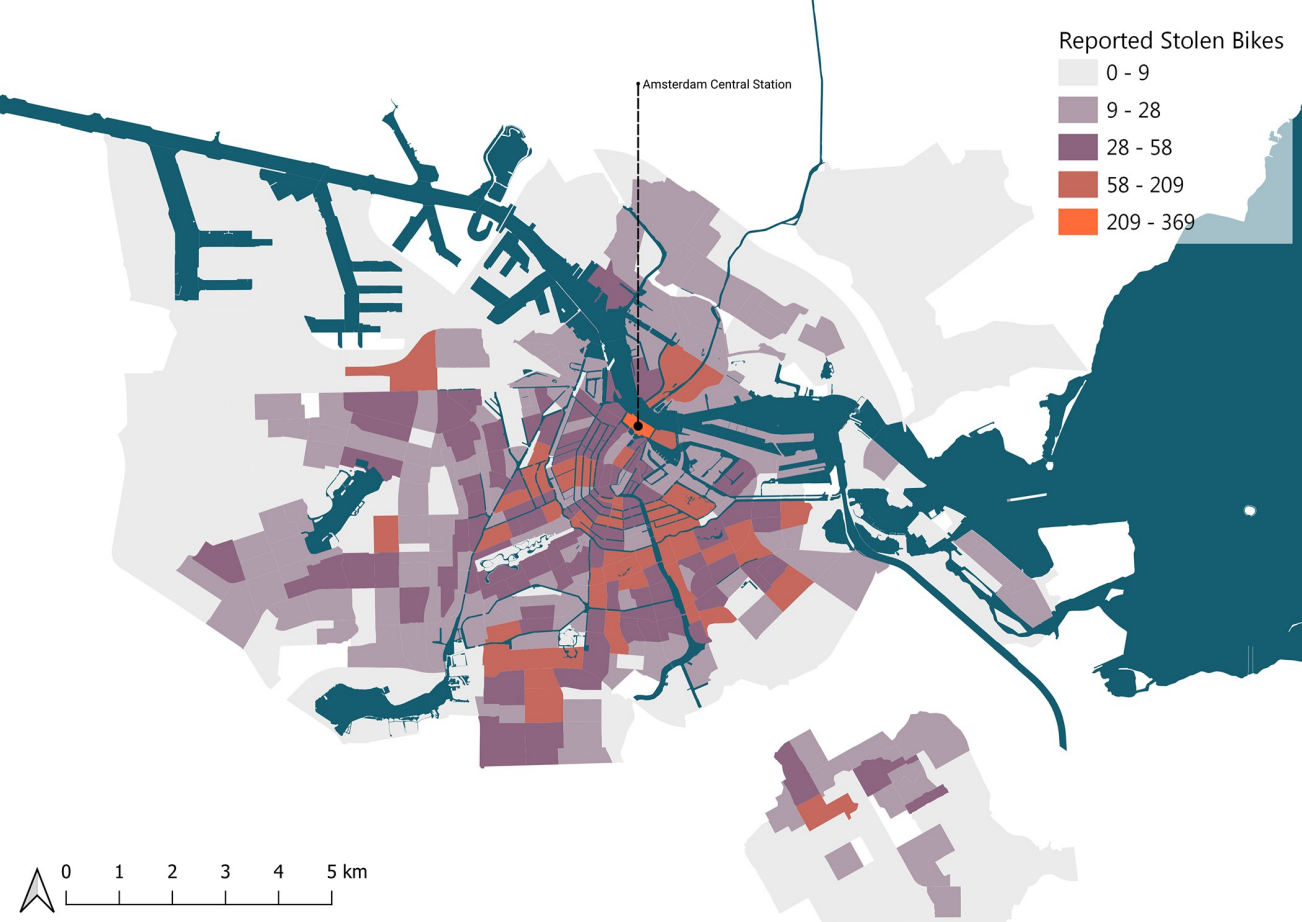
The number of reported bike thefts per neighborhood in Amsterdam (2019). Data from [15], base map data from OpenStreetMap.

Within the municipal boundaries of Amsterdam, bike thefts are not homogeneously spread. Amsterdam counts ± 470 neighborhoods, as demographic and bike theft statistics are known at this administrative level on the city, this geographic scale is suitable for analysis. Bike theft hotspots can be identified on this neighborhood level as shown in Fig 2, with the main train station, Amsterdam Central, as the top hotspot.

Some aspects of the cycling culture in Amsterdam are important to be taken into consideration: Nello-Deakin and Nikolaeva [18] interviewed 28 expats living in the city and found that the easy and inexpensive access to bikes contributes to people taking up cycling. However, many of the interviewees admitted to buying bikes from dubious origins at flea markets and informal channels, mainly because those bikes are often cheaper than the ones found in stores. “For many interviewees, bicycles were considered a semi-disposable commodity, prone to being regularly discarded, replaced, or stolen (8 interviewees reported having a bike stolen)” [18(p297)]. Some of their interviewees actually indicated that they were aware of the fact the bikes they bought were stolen. One interviewee indicated she had her bike stolen 7 times in the past 1.5 years. Therefore, the willingness to buy a bike from an expensive store decreases even further. Nello-Deakin and Nikolaeva [18(p297)] conclude that “[…] this does not mean bicycle theft is an incentive to cycling in itself, but it does suggest that widespread bicycle theft–and the informal second-hand market associated to it–are a prominent symptom or consequence of the abundance of inexpensive bikes within the urban landscape.”.

The main conclusions of our literature scan are as follows: there is little reliable data about the bike theft issue, as many citizens do not report bike theft and local authorities have other priorities. However, it is clear that with the increase of bicycles in cities the aggregate costs of bike thefts are substantial. Additionally, with limited resources and tools, cities around the world struggle to curtail thefts. Therefore, this study has two main purposes: understand where bikes stolen in Amsterdam go, and investigate whether certain urban features explain where they are stolen and where they re-enter circulation. Deriving from this purpose, we also aimed to find spatial factors that determine the likelihood of a bike being stolen in Amsterdam, what the geographic area of the stolen-bike market of Amsterdam is, and whether second-hand bike stores contribute to the recirculation of stolen bikes.

## Methodology

To answer the question of where stolen bikes go, we designed an unprecedented, yet very straightforward methodology: we distributed 100 locked bicycles, equipped with location sensors, in main bike-theft hotspots in Amsterdam, and monitored these traceable bikes.

**Fig 3:**
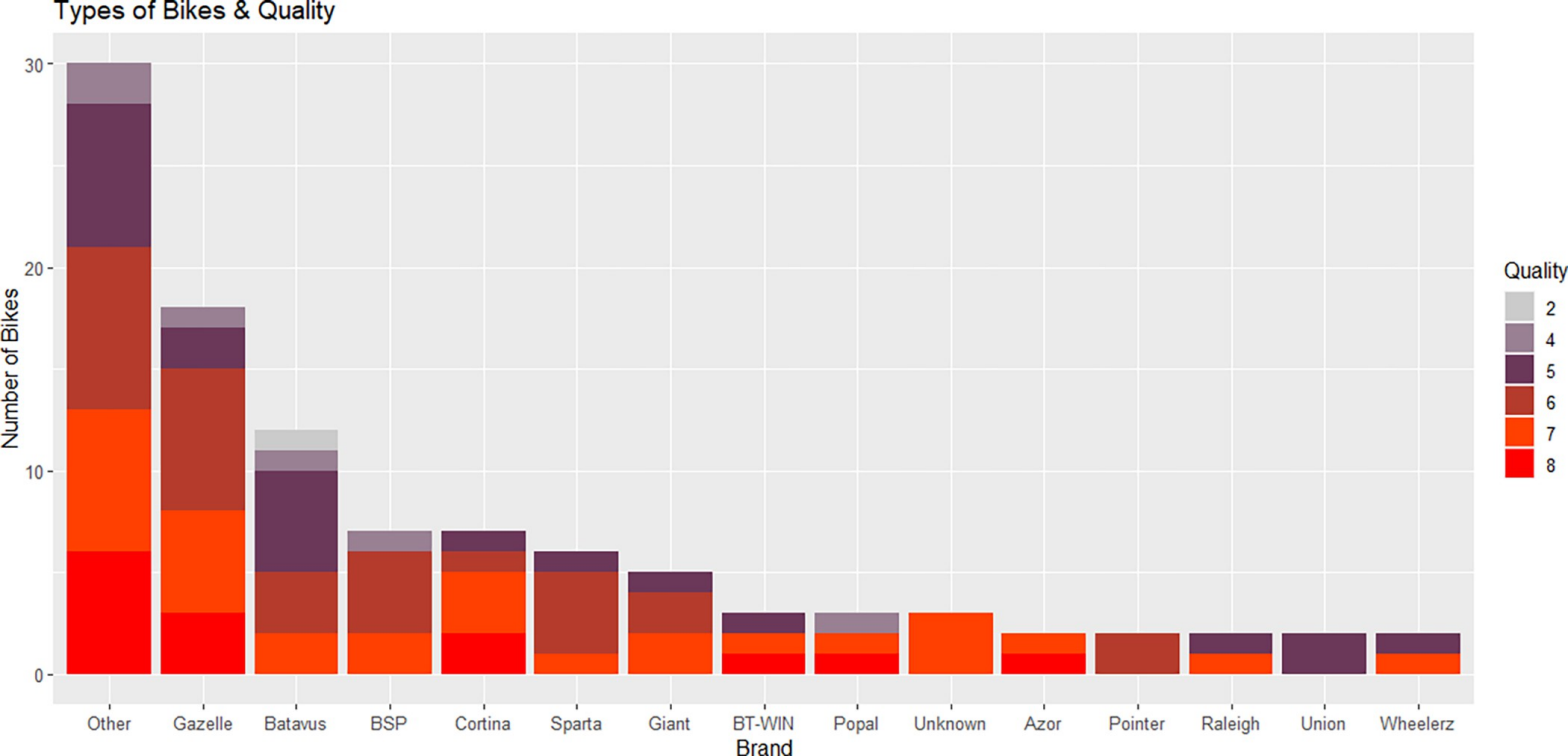
The brands of the bikes and the quality of each bike.

**Fig 4:**
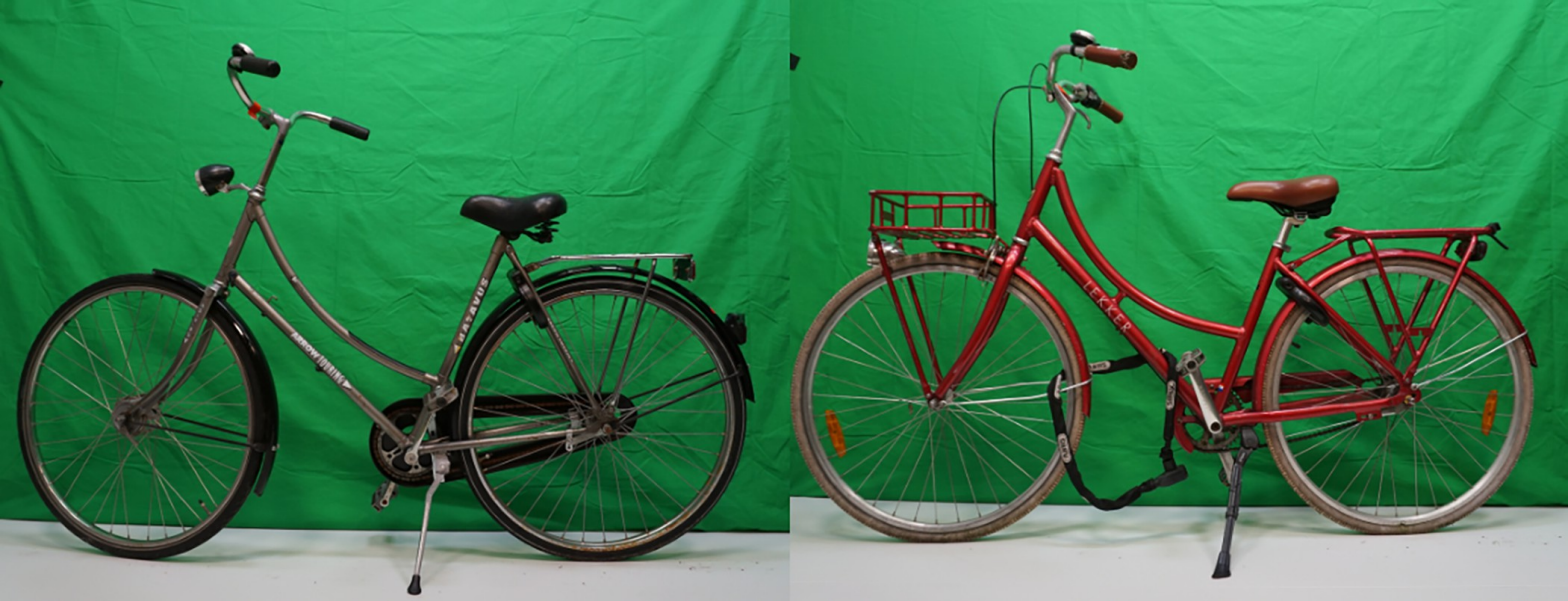
Two of the bikes used in the study, on the right an older model Batavus, on the left a newer bike from the brand Lekker.

**Fig 5:**
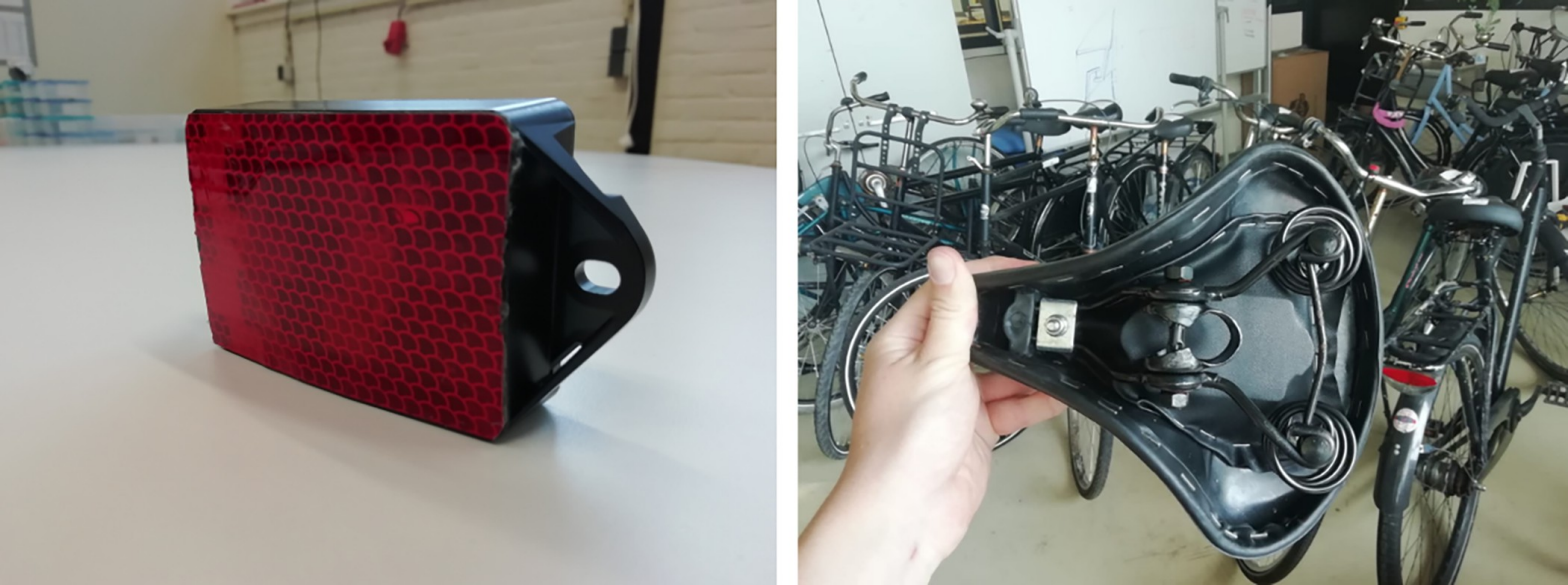
left: Placement of tracker as reflector, right: Placement of tracker underneath the seat.

Only second-hand bikes from many different brands and conditions were included in the study. The condition of the bikes was evaluated by ranking each of the 100 bikes on their usability and appearance on a scale of 1–10, where a score of 1 indicates an almost unusable bike, with many rust markings and loose cables, a score of 10 would indicate an almost new, very well maintained bike. The various brands and conditions of the bikes are summarized in Fig 3. Many of the bikes were similar to the Batavus and Lekker bikes shown in Fig 4. The same type of lock was used in all bikes, to minimize their influence in attracting or repelling bike thieves. The used sensors are shown in Fig 5, relying on SigFox and GPS-localization, with a 3-year+ battery life. These sensors send a location update when the bike starts moving, then every 10 minutes while it is on the move, and one update when it stops for more than 5 minutes.

The deployment locations of the bait bikes were determined by using the relation between the number of bikes present in each neighborhood and reported bike thefts, and by using the influence of various amenities in each neighborhood on the number of bike thefts. A spatial grid was constructed by first identifying which selected features in the built environment influence bike thefts and then using weighted kernel density estimation to identify areas with a high density of these features.

One challenge to investigate whether bike thefts are related with the number of bikes present in a neighborhood is that there is no official source for this number. Therefore, we used a computer vision model to quantify the number of bikes on street view images. Computer vision (CV) is widely used for many different applications. For the urban context, CV enables the analysis of large amounts of streetscapes on an extremely fine grain scale. This can provide insights into how streets are perceived [19] or how streets are used based on their desirability [20]. In this paper, the focus is on getting an estimate of the number of bikes present in each neighborhood of Amsterdam. This was done by automatically counting the number of bikes in images using a panoptic segmentation method. This method relied on a pre-trained image processing model developed by Facebook’s AI Research (FAIR) group, which is part of the Detectron2 object detection platform [21]. The Detectron2 platform incorporates a method referred to as Panoptic Segmentation FPN, which was used to count the number of instances labeled as a bike [22].

The Detectron2 platform includes three panoptic segmentation models relying on the COCO (Common Objects in Context) data set [23], which are compared using several baselines [21]. Based on these baselines, the R101-FPN model was selected as the Panoptic Quality metric performs best.

**Fig 6:**
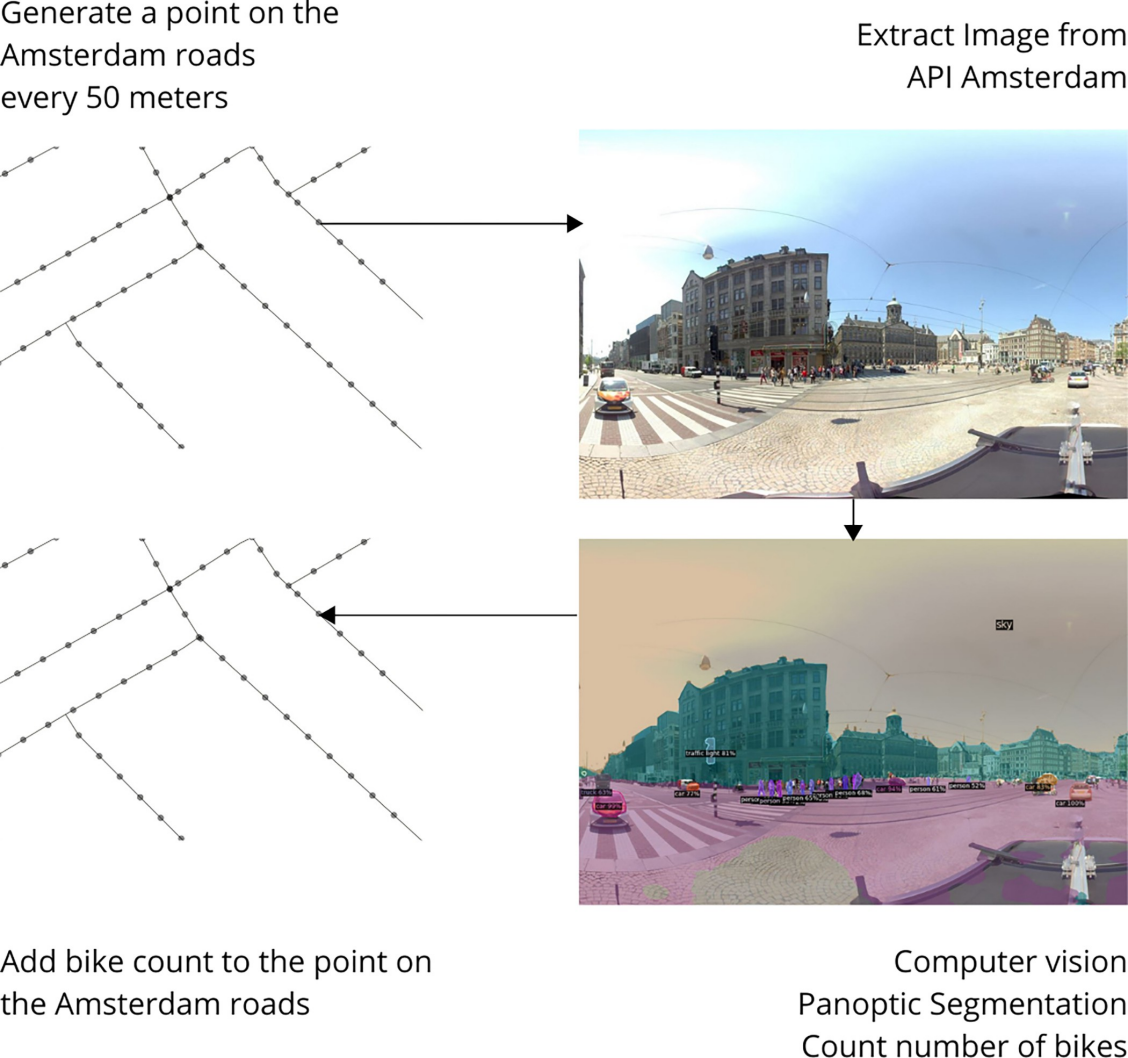
Schematic of the panoramic image processing. Original images: Municipality of Amsterdam.

The image analysis was initiated by using a dataset of the entire road network of Amsterdam, based on the dataset Wegen (“Roads”) from Open Street Map [24], which was overlapped with the municipal boundaries of Amsterdam. On this road network, points were generated on the lines with a distance of 50 meters from each other. 50 meters was chosen as distance to maximize the possibility of getting an image, but limit the chances of having duplicated images. For each point on the road network, the latitude and longitude were extracted and forwarded to the panoramic image API (Fig 6). This collects the panoramic image closest to the latitude and longitude of the points with a buffer of maximum 40 meters to ensure that we do not have overlapping images.

Each image, connected to one of the ~82,000 points in Amsterdam, was subsequently analyzed using the selected pre-trained panoptic segmentation model. This process results in points with an attribute indicating the number of bikes in each image (Fig 6). These points were subsequently loaded into QGIS and aggregated to the neighborhood level, by counting the number of points in the neighborhood polygons with a weight factor equal to the number of bikes visible in the images. After this process, the number of bikes per neighborhood was divided over the number of points per polygon, resulting in the average number of bikes in each image per neighborhood.

**Fig 7:**
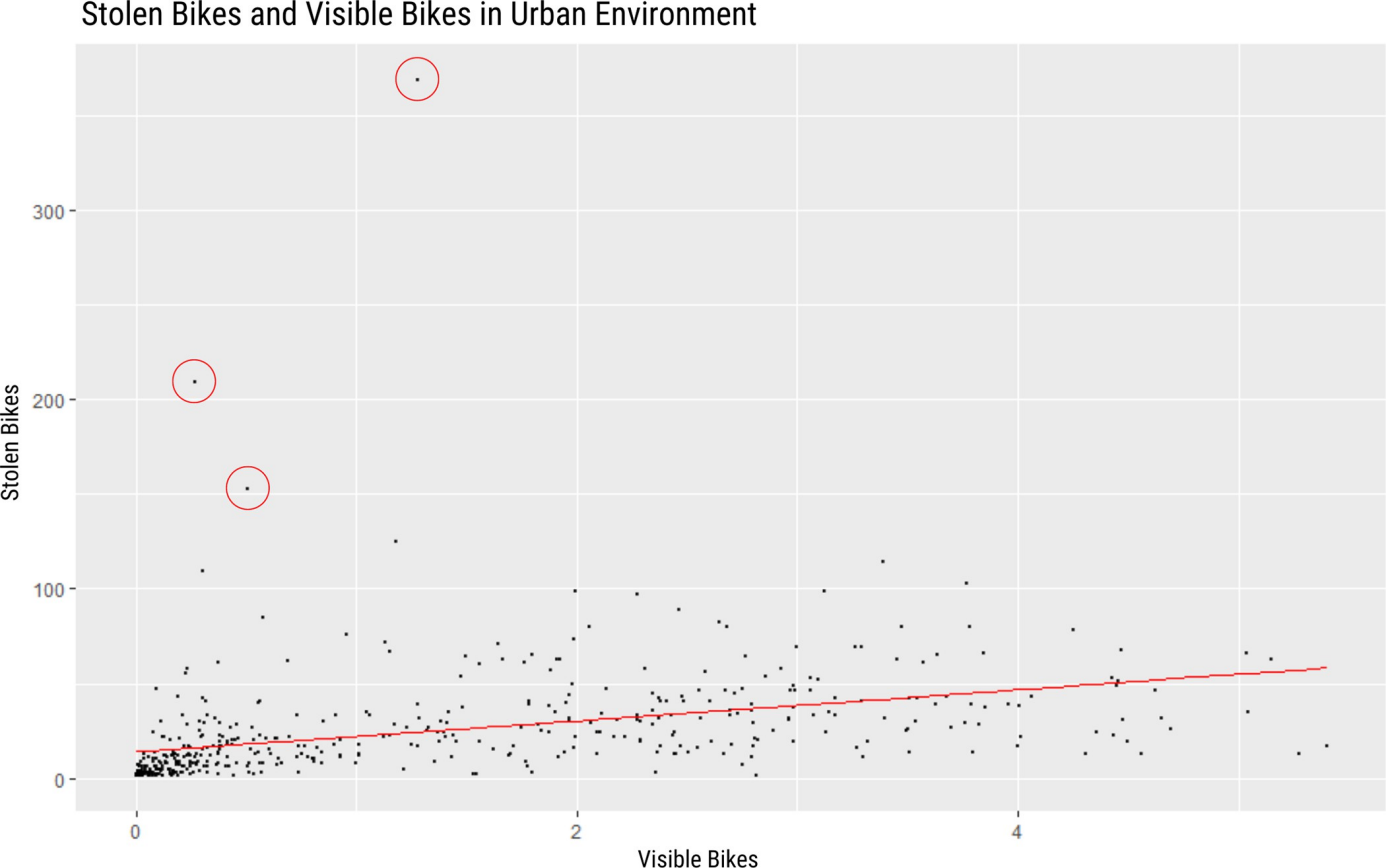
Stolen bikes and average number of bikes in the images. The linear model is represented by the red line.

In the next step, data about the frequency and location of bike thefts were collected from the Dutch National Police [25]. This data consists of the number of bike thefts per neighborhood (n = 470) in Amsterdam. The average number of bikes per image was compared with the number of thefts per neighborhood using linear regression, as visualized in Fig 7. The highlighted outliers with the red circles are areas with public transportation facilities that have bike parking which might not be visible from the roads. Therefore, the actual number of bikes should be higher than the estimate presented here. The regression analysis showed significant results (p < 2e-16). Therefore, the number of bikes per neighborhood appears to have a substantial influence on the number of stolen bikes.

To identify amenities that influence the number of bike thefts, we collected OpenStreetMap data [24], which includes several categories of amenities represented by points of interest, such as schools, restaurants, and train stations. The number of specific amenities per neighborhood was used as input in a multiple linear regression (MLR) model to predict the number of bike thefts. This approach is similar to the study of bike theft in London by Mburu and Helbich [26] to investigate which spatial characteristics influence the bike theft incidence.

Based on this MLR and ANOVA type II test, the following features are significantly influencing the bike theft occurrence in Amsterdam: the number of bikes in a neighborhood (measured using the image analysis), bike stores, homeless shelters, supermarkets, cafes, schools, train stations, taxis, tram stops, and bus stops.

**Fig 8:**
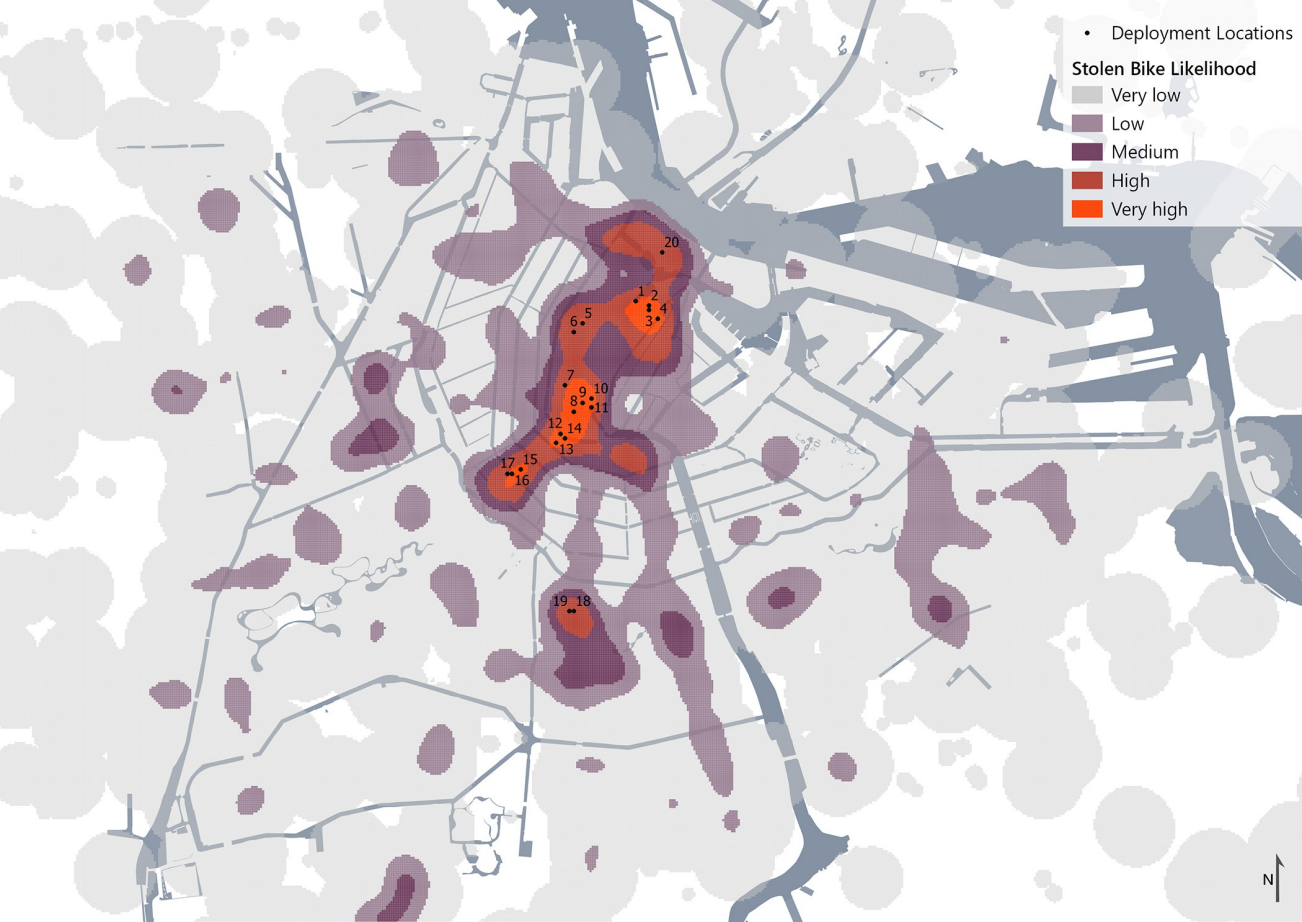
Deployment locations and likelihood indicator, base map data from OpenStreetMap.

These findings were translated into a likelihood indicator for bike theft using weighted Kernel Density Estimation, where the significance of the relation and the correlation coefficient were used as weights. To ensure a diverse range of deployment locations, the 20 neighborhoods with the highest likelihood were selected. Within these neighborhoods, the point with the highest likelihood was selected. This resulted in the following deployment locations (Fig 8).

**Fig 9:**
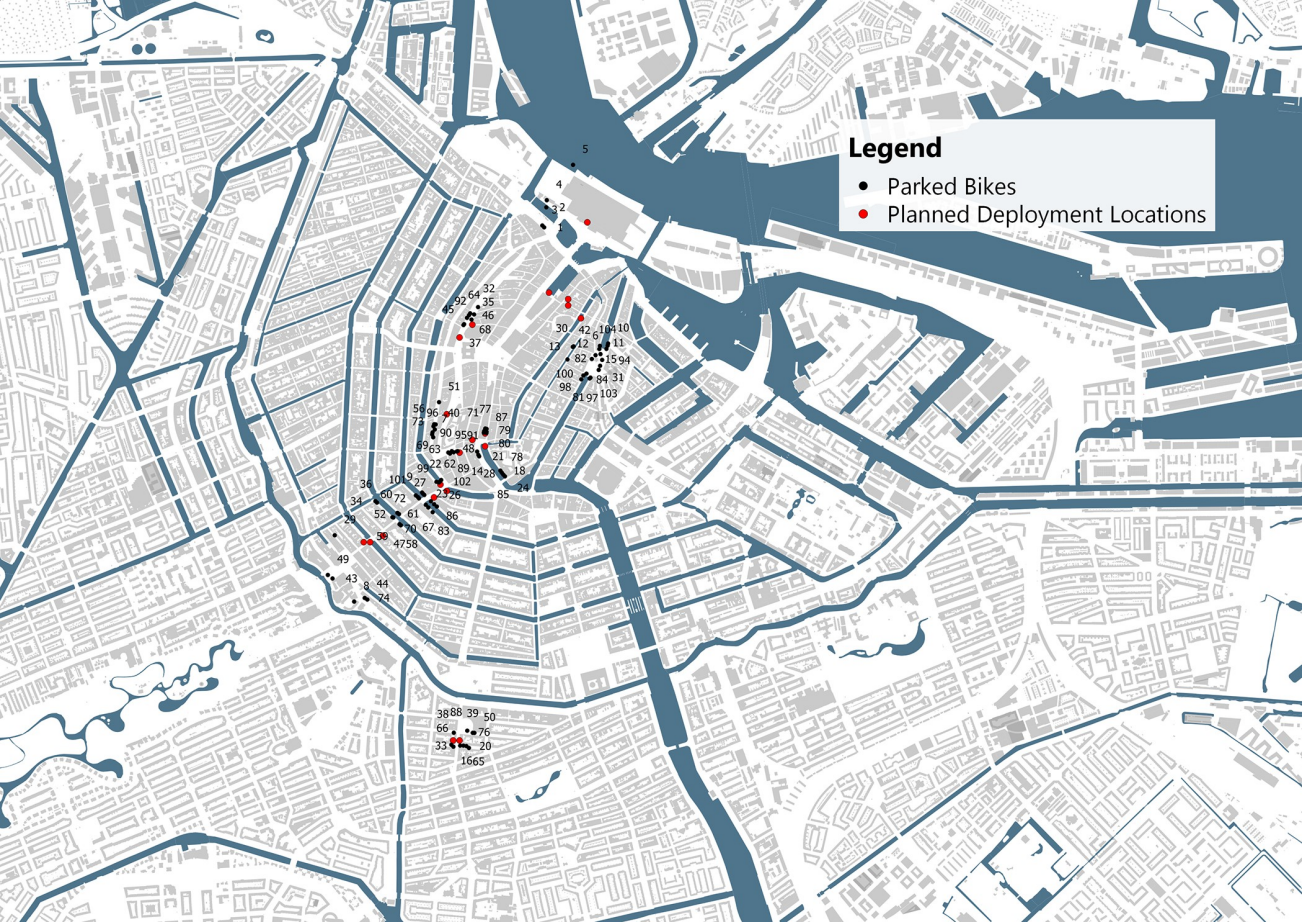
Planned deployment locations and realized parking locations of the 100 bikes. Base map data from OpenStreetMap.

The bikes equipped with sensors were deployed in these locations. Each bike was locked either with the lock looping through the front or back wheel, or the bike was attached to bike parking infrastructure, if this was available on the deployment location. There were minor deviations from the determined deployment locations, as shown in Fig 9, due to practical constraints such as road closures and pedestrianized zones.

Between the 1st of June and the 30th of November 2021, the 100 deployed bikes were tracked. During this period, 70 bikes were classified as stolen, with a specific date and time when the theft occurred. Each of the 70 stolen bike routes were then analyzed to determine when the bike started to be used regularly again following repetitive spatial and temporal patterns (frequent visits to same locations, from a same origin)—which would indicate commuting, at which point we stopped the analysis to avoid risking identifying the user (which is not the goal of this research and could entail privacy concerns).

Due to interference and poor SigFox coverage the reported locations while the bikes were moving were removed. This resulted in a clean dataset with only the start and stop points of the trips of the 70 stolen bikes.

The geographic area of a stolen goods market could be used as a boundary condition to develop strategies and policies to reduce theft. The used method of analysis is straight forward: the number of visits of a stolen bike to predefined areas is counted. In this case, we use the geographic units of 4-digit postal codes, which provides more detailed information than neighborhood levels, yet ensuring that no individuals or organizations can be identified.

One of the first hypotheses we tested was whether second-hand bikes stores had any role in the recirculation of stolen bikes. To do that, we calculated the straight-line distance from the stop locations of the 70 stolen bikes to the nearest bike store in the Netherlands. If these stop points were within 50 meters of a bike store, they were flagged for further analysis. Additionally, the time spent at these stop points was used to see how long these bikes remained parked at a bike store, omitting visits shorter than one hour as a bike store cannot assess, repair, and sell a bike in under an hour. The stolen bike routes with at least one flagged stop point were manually inspected further to investigate the movements of the bike before and after the visit to the bike stores. If these routes exhibited a commuter pattern after the potential visit to the bike store, but not before, the bike was counted as “sold at a second hand bike store”. For some stolen bikes, the tracker was permanently disabled during the potential visit to the bike store, which was also flagged as “sold at a second hand bike store”.

To identify relevant typologies of stolen bike routes, the following characteristics about the routes and the bikes were collected as shown in Table 1. Firstly, k-means was used to identify clusters in the data, after which clustering algorithms such as dbscan and hierarchical clustering were used.

**Table 1:** Collected variables of the stolen bikes’ routes and characteristics.

Abbreviation	Description
Distance_tot	total distance traveled
Distance_av	average distance between start and stop points
Distance_longest	distance of the longest trip of the bike
Speed_av	average speed
Speed_max	maximum speed of the bike
Stolen_time	the amount of time in hours between theft and re-entry to market
Points	amount of start and stop points between theft and residential pattern
Bike_store_min	minimal distance to a bike store
Condition	State of the bike (e.g. rust, loose cables) as evaluated by researchers, scale 1 to 10.
Gears	amount of gears on the bike
Unique visits	amount of new locations the bike visits (based on 40 meter radius)
Visits_max	amount of visits to the most visited location
Visits_mean	average amount of visits to a location
Av_start	average amount of start points of a bike

Network analysis was used as another approach to identify similar routes. A network was created consisting of bikes and locations. This was done by creating a grid of 500 by 500 meter cells over all the locations the stolen bike traveled to. This enables routes to be described as “bike 1, from cell 30 to cell 25”. As the bikes were deployed in groups, the deployment locations were omitted in this analysis to ensure this would not influence the grouping of bikes. This was done for all the stolen bikes, resulting in a network of locations and bikes.

First, important locations for the flow of bikes in the network were identified utilizing network betweenness. The aim was to identify where stolen bikes travel to, and which locations might be important without having many amenities or functions in the area. Locations with many functions would result in bikes visiting these areas more often naturally, while locations without any functions should have very few or no bikes visiting it. If there are locations without many amenities that are important for the flow of stolen bikes, there might be information about the network available to the people stealing the bikes, this could either mean organized crime or each individual has the same knowledge about how to sell a stolen bike.

Second, community structures within the network were investigated using the multi-level optimization of modularity approach for finding community structure as implemented in the Igraph R package [27]. This identifies communities within networks and can, therefore, connect bikes and locations to each other if they have overlapping characteristics.

## Results

**Fig 10:**
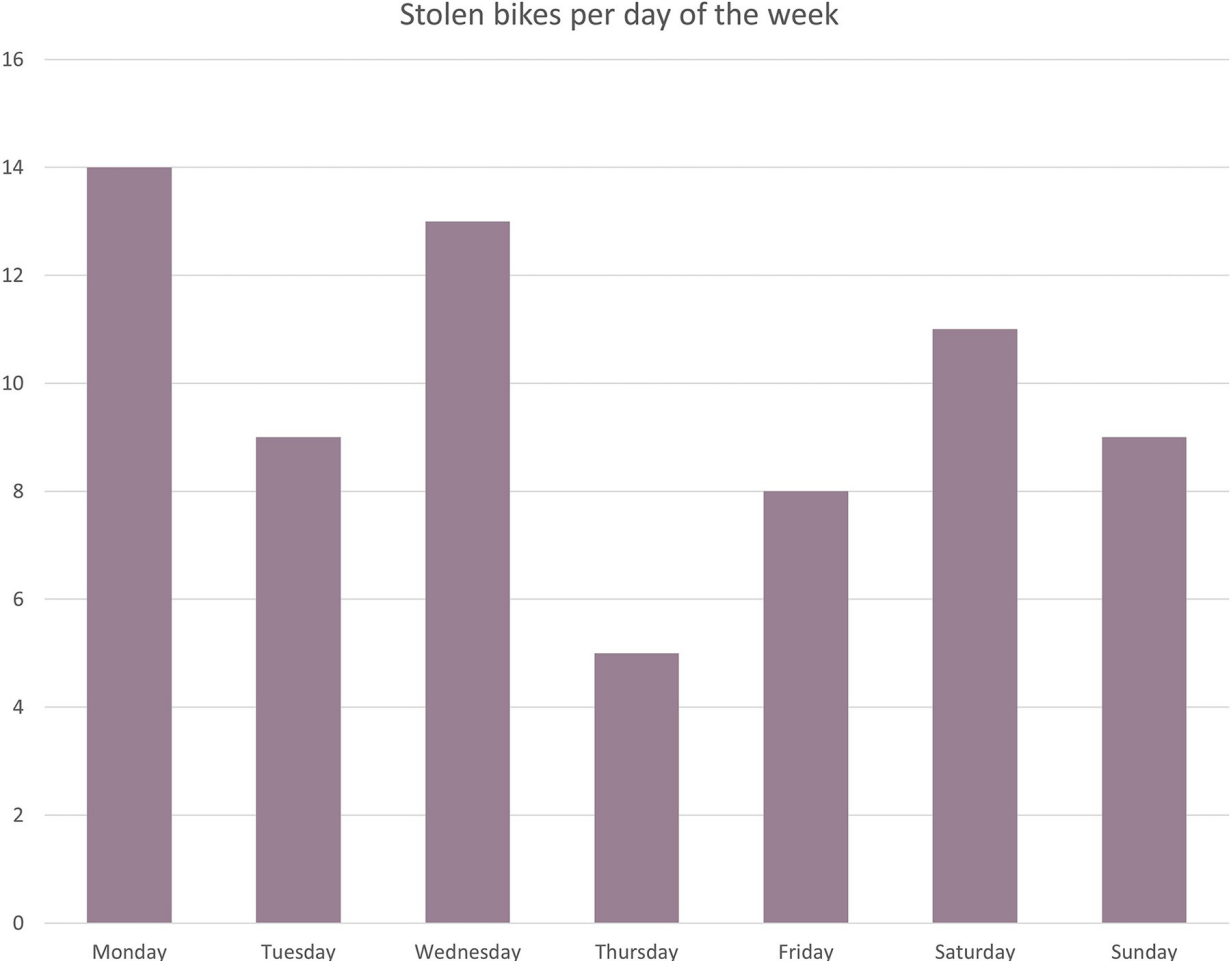
Count of the number of bikes stolen per day.

**Fig 11:**
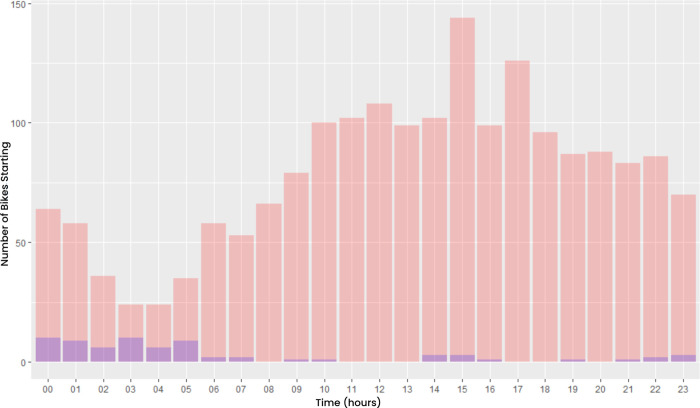
Count of the number of start times of a trip per hour. Purple indicates the start time of the first trip, right after it was stolen.

**Fig 12:**
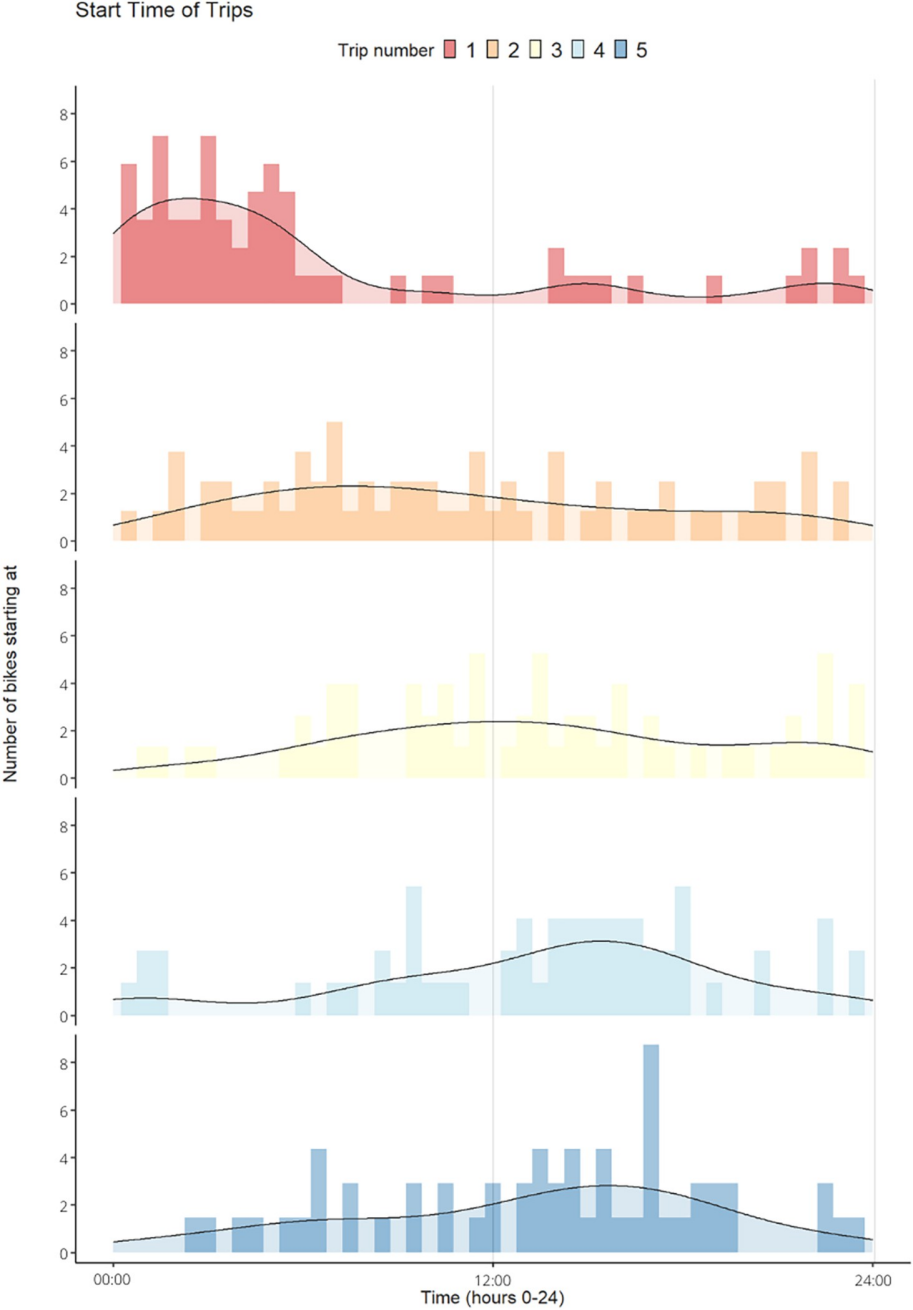
The number of bikes that started at the same time (could be a different date) for each of the first five trips the stolen bikes made. The start times of trip one are the times the bikes were stolen at.

Exploratory data analysis showed that bike theft was most likely to occur on Mondays and Wednesdays (Fig 10), mainly during the night around 03:00 AM. While comparing the time when the bikes were stolen to the start times of all the trips the bikes took, as shown in Fig 11, a pattern similar to day-to-day mobility can be identified. Therefore, the first 5 trips of all the bikes and their start times were investigated further. This resulted in the conclusion that around the fourth trip (Fig 12), most bikes were moving during the daytime as one would expect with a mobility pattern. This indicates that bikes remain “stolen” for a relatively short time, quickly returning to a regular pattern of bike mobility.

**Fig 13:**
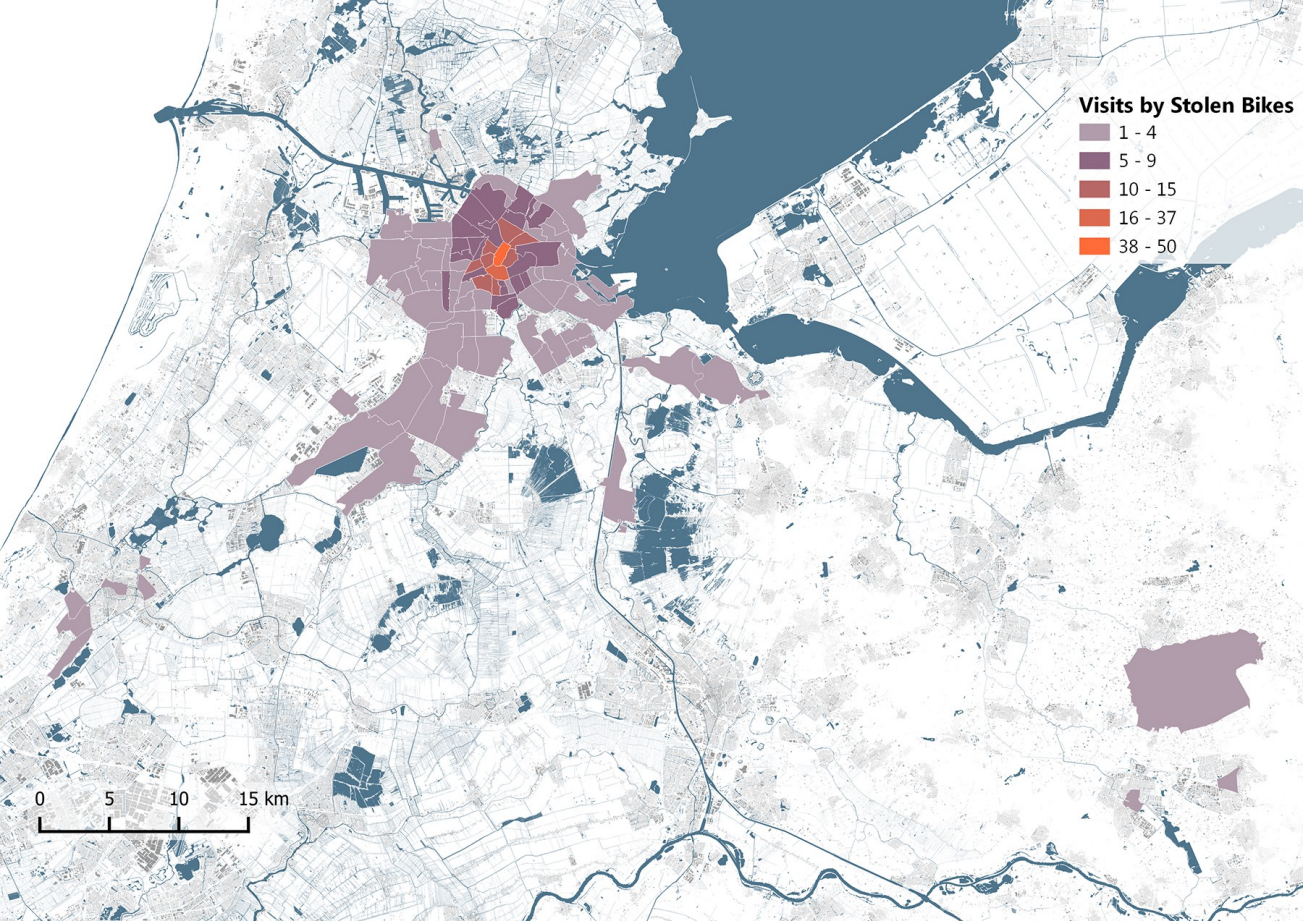
Visits of stolen bike to 4-digit postal code, base map data from OpenStreetMap.

The geographic area of the stolen-bike market is shown in Fig 13. Only two bikes out of the 70 have moved substantially beyond the municipal borders of Amsterdam. Therefore, it can be concluded that the stolen-bike market is very local.

Based on the analysis, 3 to 6 bikes exhibit a pattern associated with retail in a second-hand bike store. If we assume the sample to be representative of the larger group of stolen bikes, this would mean around 4.3% - 8.6% of stolen bikes are sold through official bike stores. This raises the question whether these bike stores knowingly participate in selling stolen property. Further research could address this issue, as in this paper we have not assessed whether bike stores followed specific procedures to identify a stolen bike presented as a second-hand bike, and approaching them would breach privacy securities we ought to maintain. Here we should assume that second-hand bike stores might be participating in the stolen-bike market unwillingly.

**Fig 14:**
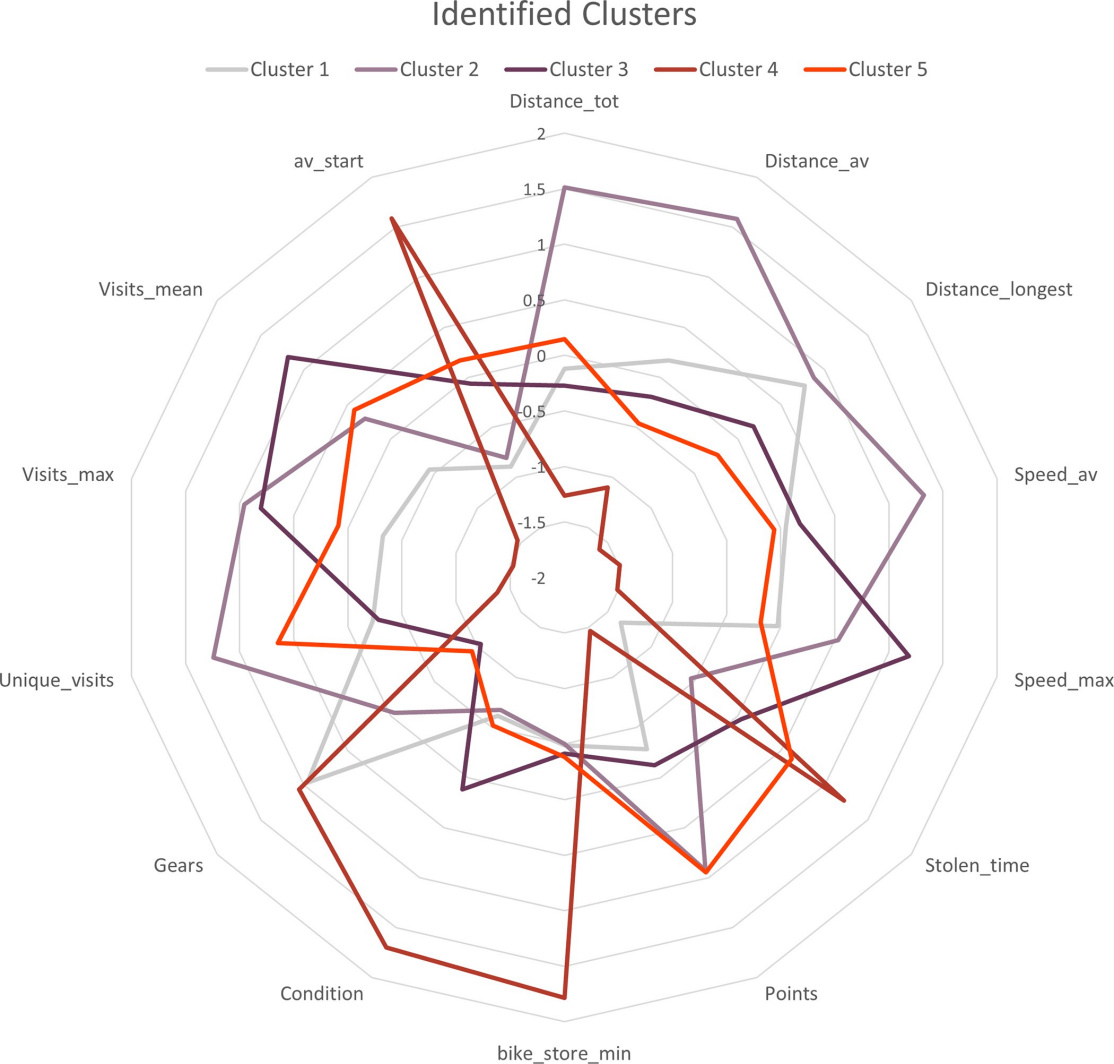
Hierarchical clustering results, visualized as the deviation of the cluster from the overall mean providing information about the differences between all clusters.

Several characteristics of each bike and the stolen bike’s route (Table 1) were aggregated to one dataframe. Several clustering techniques were used, among which k-means, dbscan, and hierarchical clustering. The results of the hierarchical clustering seemed most promising (Fig 14); however, as unsupervised clustering has its limitations with regards to interpretability, this method did not yield relevant information about potential networks or groups of bikes with similar stolen-bike route characteristics.

The network analysis was initiated by generating grids of 500 by 500 meter cells over all the visited 4-digit postal codes in the Netherlands. Subsequently, a network was created by adding the id number, location and time attributes of each bike’s location to the cells. Then a network was created and the betweenness of each location was calculated. The betweenness indicates how often the location is on the shortest path between other locations, the larger the betweenness the more important this location is to the flow of stolen bikes in the network.

**Fig 15:**
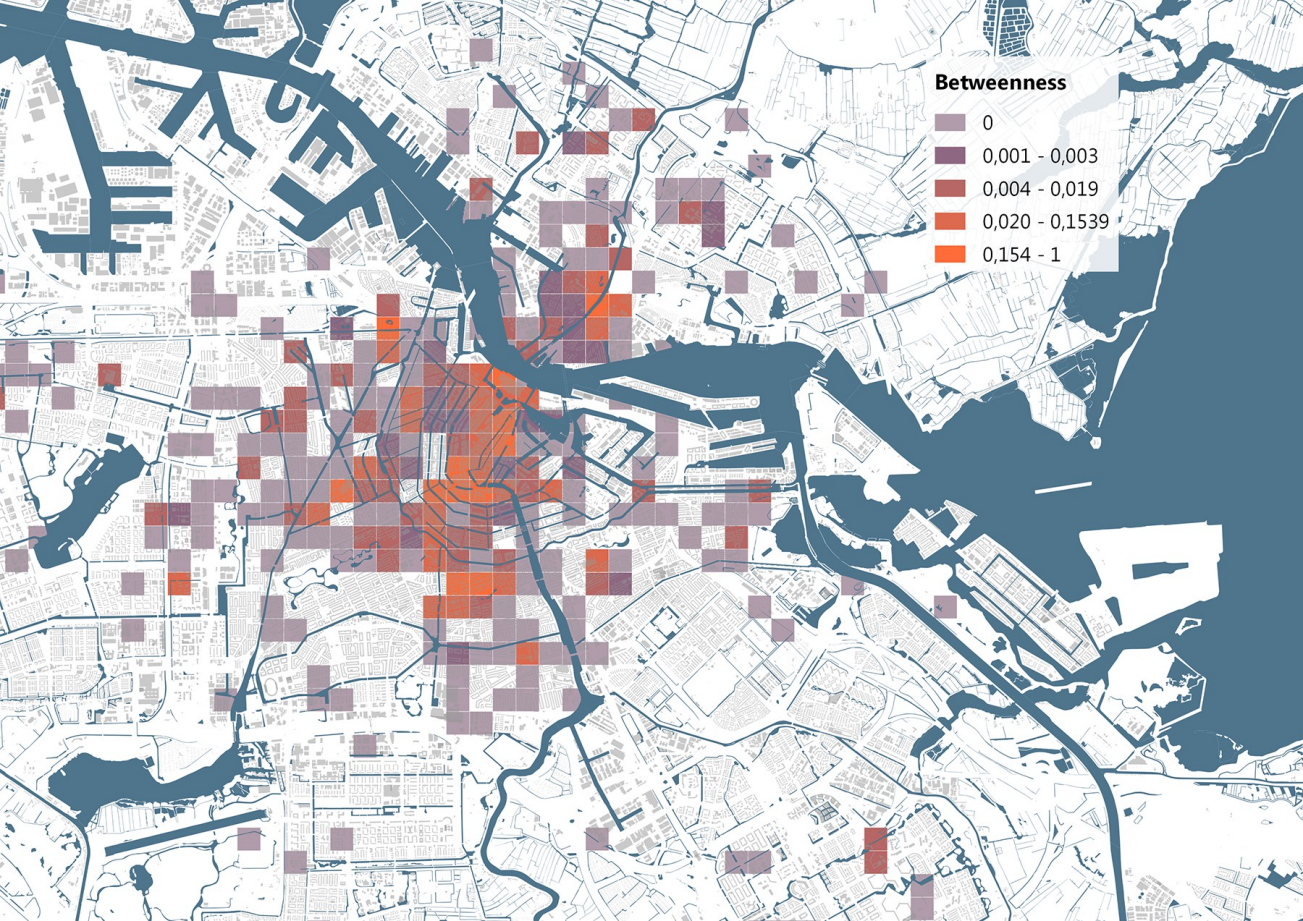
Betweenness, and therefore importance for the movements of stolen bikes, of the grid cells. Base map data from OpenStreetMap.

**Fig 16:**
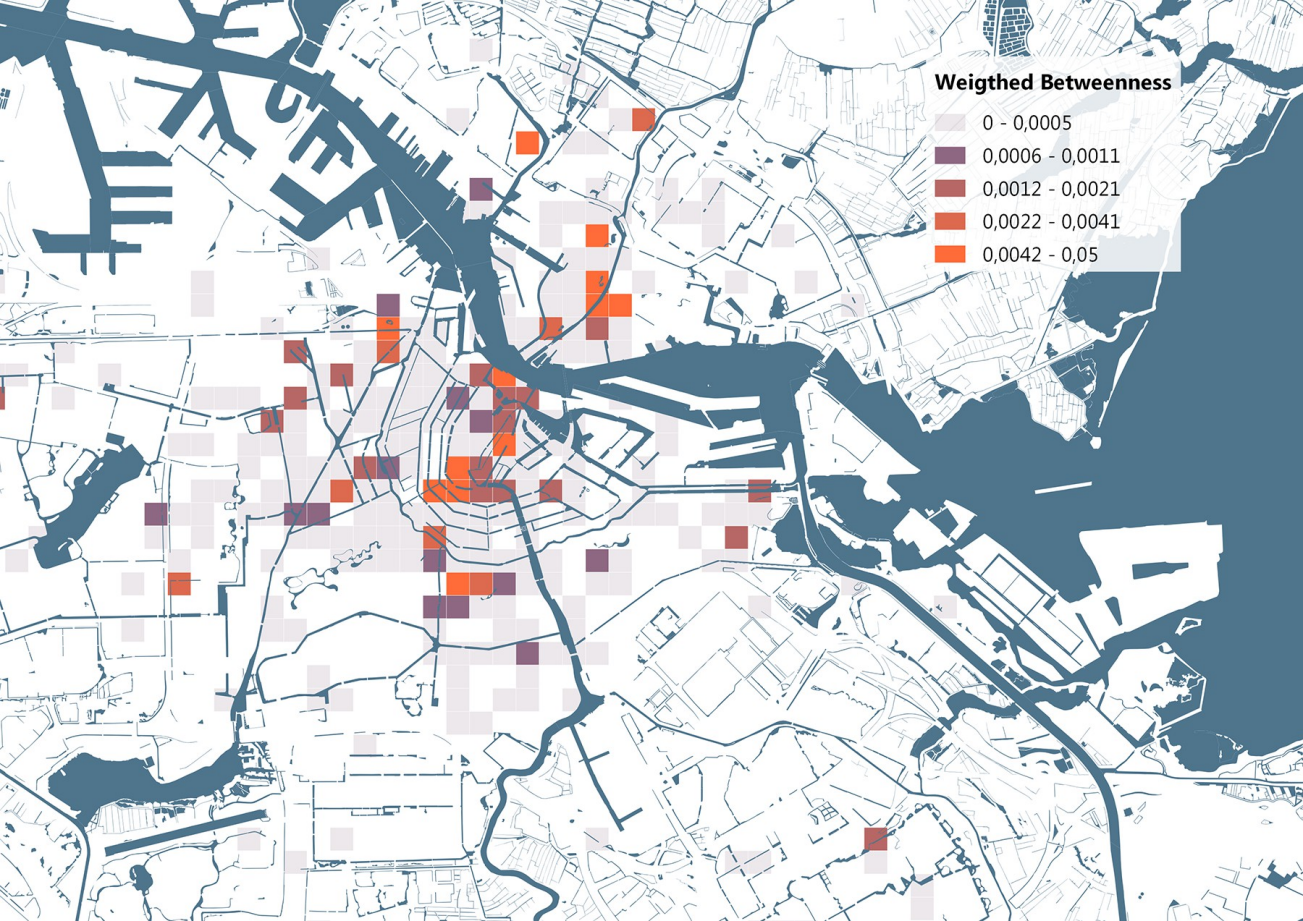
Weighted betweenness identifying grid cells with few functions and amenities yet a high importance for the movements of stolen bikes. Base map data from OpenStreetMap.

The betweenness measure of each location was visualized on the map, as shown in Fig 15. To identify areas in the city with an unusually high flow of bicycles, we attempted to adjust the betweenness measure by using the presence of amenities as a weight factor. For each grid cell we summed the number of points-of-interest included in OSM data, this dataset spans a wide range of amenities from park benches to restaurants to train stations. We then divided the betweenness measure over the total number of these points-of-interest, as these points should make those areas more important in the network, this resulted in the weighted importance which is visualized in Fig 16. Based on the identified areas, we investigated whether specific bikes are responsible for why these areas are important, and what the connections might be between these locations.

**Fig 17:**
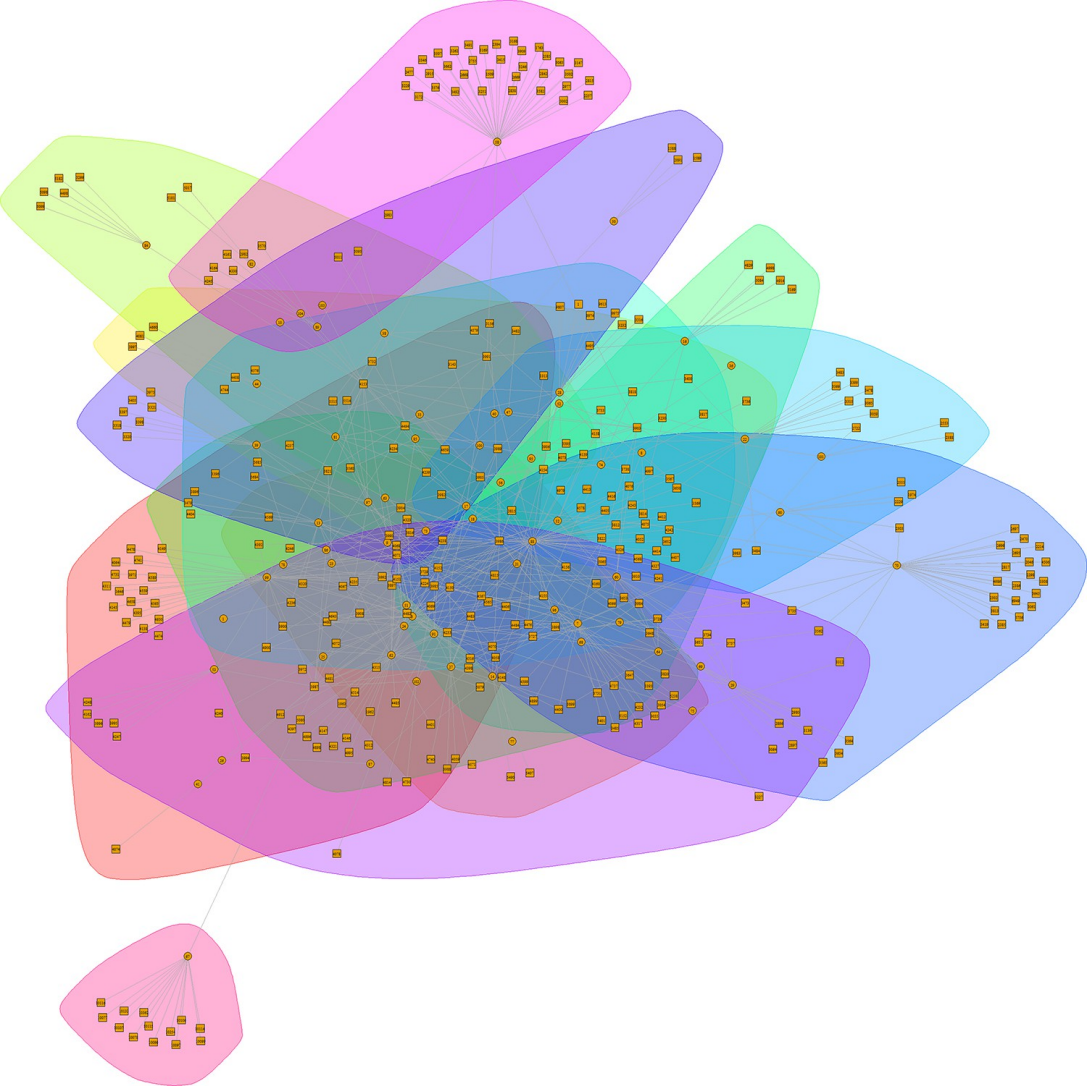
Identified communities of stolen bikes based on the Louvain algorithm.

To identify communities or linked bicycles and locations in the network, a community analysis was performed. Specifically, the Louvain algorithm was used, which implements the multi-level modularity optimization algorithm for finding community structures.^27^ The Louvain algorithm can cluster networks based on links within a network. It does this by optimizing modularity, which is a measure of comparison between links within communities or between communities in networks. The visualization illustrates the generated network, and it colors which nodes and links are clustered into which community within this network. The identified communities are shown in Fig 17. This analysis uncovered 13 communities of bikes, based on a standard gamma value of 1. These communities were manually investigated further. This revealed that 22 out of the 70 stolen bikes were linked in a subnetwork. From this, it can be concluded that around 30% of the stolen bikes seem to be stolen by organized crime or by offenders with a lot of knowledge about the overall bike theft system. Additionally, 12 bikes visited known locations where stolen bikes are frequently resold in Amsterdam, possibly indicating that around 17% of stolen bikes are resold in these places.

## Discussion

The results indicate that some amenity types have a significant influence on bike theft. This could guide the location of interventions and identify priority areas for combatting bike theft. However, it should be noted that those amenities do not generate bike thefts. The congregation of people at specific locations induced by those amenities, resulting in the availability of targets, is a likely explanation. However, the variables that induce bike theft were quantified by utilizing the locations of amenities. In this way, the amenities can be seen as a proxy measurement for all variables influencing bike theft. Additionally, the research on the effect of the availability of targets on bike theft was investigated by automatically labeling images using computer vision. As computer vision is not perfect, we can reasonably assume some misquantification of the number of available bikes. It is assumed that these errors occur regularly and therefore should not influence the relative number of bikes. Despite this, an absolute baseline was not established, therefore, there remains some uncertainty in the method of measurement.

The second part of this research investigated the deployment of 100 traceable bikes. This novel methodology demonstrates the utility of such traceable objects to shed light on movements of stolen property. The results imply that bike theft–for this segment of bikes–is mainly a local phenomenon, with a quick return-to-market, and some organized characteristics. However, it also poses legal and ethical questions. These questions were addressed systematically, and approved by the institutional review board. Despite this, with evolving legislative and ethical frameworks, the delineation of what is desirable within research will remain an important question.

Finally, there is a question about the reliability of the collected data. Some trackers were disabled at second hand bike stores or they discontinued due to unknown effects. The latter happened two times before a residential pattern was identified, as such, of these two stolen bikes little is known about the movements. For the disabled trackers, one could speculate that more organized thieves check the bikes more thoroughly before transport and identify the tracker. Therefore, the included data does not show any movements across borders. Whether this is the case, is up for speculation. However, as the large majority of sensors remained active until and after a residential pattern was established, bike theft remains a largely local phenomena for this segment of second-hand bikes.

## Conclusion

This research investigated the influence of specific amenities as well as the number of available bikes on bike thefts. The methodology developed in this paper revealed strong evidence that the quantity of bikes and specific amenity types in the urban environment increases the likelihood of bike thefts occurring. This implies that locations with more bikes and specific amenity types should be the priority when designing policies and updating infrastructures, such as bike parking facilities or surveillance.

Furthermore, we analyzed the pathways of stolen bikes by deploying 100 bikes to investigate bike theft using location trackers. We find that this approach provides valuable insights about the journey of bikes after theft. The resulting data can aid in designing new policies to more effectively reduce stolen good markets. It provides boundary conditions, such as spatial scale, for policy makers and police practices aiming to reduce crime. In the investigated case, it provides insight into the return-to-market of stolen items and the scale of the network, which can both be used to deter citizens from purchasing stolen property, as well as inform police practices directly.

For the context of Amsterdam this research shows that stolen bikes remain close to the city. The movements of the bikes indicate a wide variety pathways towards being regularly used again. We find that 30% of stolen bikes might be due to organized crime and that around 4.3% - 8.6% of bikes are resold through second-hand bike stores. By combining the insights generated by this method, we can answer the question of where stolen bike go in Amsterdam; not very far, but the way they get there is highly varied and sometimes with earlier unknown distinct patterns.

The collected data can also serve as a starting point to develop theories about the movements of stolen property. We conclude that the traveled distance of a stolen bike is influenced by where the demand is located and the potential reward. In the case of Amsterdam, the market demand for cheap bikes from dubious origins remains high. Therefore, a theory about the movements of stolen property could include locations of market demand, potential reward, difficulty of transport, and perceived risks of arrest.

In sum, we find that this approach extends the current urban crime literature with a more detailed perspective on what happens after a theft, something that had previously only been studied based on car theft locations and retrieval points [28]. The addition of cheap and easily available digital technologies enables new research into this field and can provide opportunities for municipalities to reduce crime rates. Therefore, with the rise of bicycles in cities increasing the number of bike thefts, tracking technology can be a great asset to increase safety, inform policy, and design police practices around the world.

## Supporting information

S1 Appendix Table with statistics on the various amenity types included in the research. (DOCX)
