# Supplementary material for: Tracking stolen bikes in Amsterdam
Source: PLoS One. 2023 Feb 15;18(2):e0279906. doi: 10.1371/journal.pone.0279906 (PMC9931151; doi:10.1371/journal.pone.0279906)

# Appendix

Appendix 1: Table with statistics on the various amenity types included in the research.


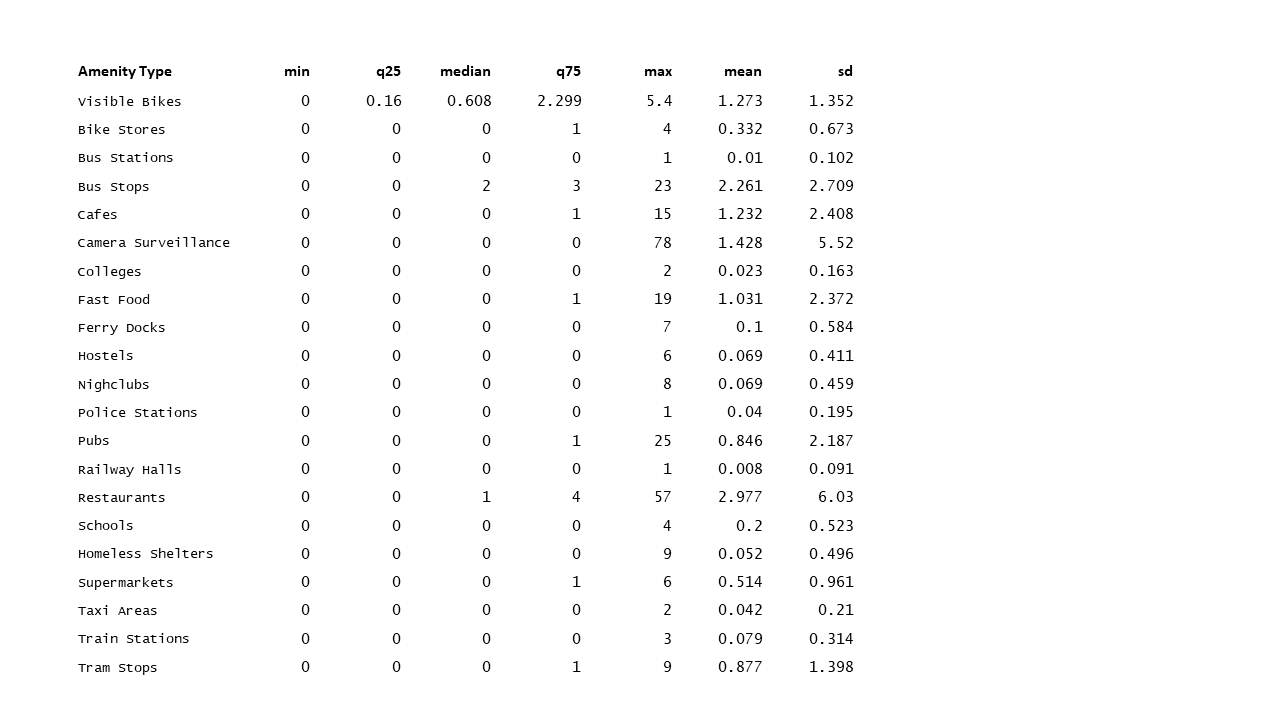

Supplement: S1 Appendix — (DOCX) [file pone.0279906.s001.docx]
